# Modelling the influenza disease burden in people aged 50–64 and ≥65 years in Australia

**DOI:** 10.1111/irv.12902

**Published:** 2021-09-29

**Authors:** Aye M. Moa, Robert I. Menzies, J. Kevin Yin, C. Raina MacIntyre

**Affiliations:** ^1^ Kirby Institute University of New South Wales Sydney New South Wales Australia; ^2^ Medical, Sanofi Pasteur Australia and New Zealand Sydney New South Wales Australia; ^3^ Medical Department of Global Influenza Franchise Sanofi Pasteur Singapore; ^4^ Faculty of Medicine and Health University of Sydney Sydney New South Wales Australia

**Keywords:** hospitalisations, influenza, influenza mortality, modelling, myocardial infarction, respiratory

## Abstract

**Background:**

Estimation of influenza disease burden is necessary to monitor the impact of intervention programmes. This study aims to estimate the attributable fraction of respiratory and circulatory disease due to influenza among Australian adults 50–64 and ≥65 years of age.

**Methods:**

A semi‐parametric generalised‐additive model was used to estimate annual and average rate of influenza‐attributable hospitalisation and death per 100,000 population under the principal diagnosis of influenza/pneumonia, respiratory, circulatory and myocardial infarction (MI) from 2001 through 2017.

**Results:**

Over the study period, seasonal influenza accounted for an estimated annual average respiratory hospitalisation rate of 78.9 (95%CI: 76.3, 81.4) and 287.5 (95%CI: 279.8, 295.3) per 100,000 population in adults aged 50–64 and ≥65 years, respectively. The corresponding respiratory mortality rates were 0.9 (95%CI: 0.7, 1.2) and 18.2 (95%CI: 16.9, 19.4) per 100,000 population. The 2017 season had the highest influenza‐attributable respiratory hospitalisations in both age groups, and respiratory complications were estimated approximately 2.5 times higher than the average annual estimate in adults aged ≥65 years in 2017. For mortality, on average, influenza attributed 1,080 circulatory and 361 MI deaths in adults aged ≥65 years per year. Influenza accounted for 1% and 2.8% of total MI deaths in adults aged 50–64 and ≥65 years, respectively.

**Conclusion:**

Rates of cardiorespiratory morbidity and mortality were high in older adults, whilst the younger age group contributed a lower disease burden. Extension of influenza vaccination programme beyond the targeted population could be an alternative strategy to reduce the burden of influenza.

## 1 BACKGROUND

People of all ages are susceptible to influenza infection. In Australia, annual influenza vaccination is recommended for people from 6 months of age and is available under the National Immunisation Program (NIP) for adults aged 65 years and over and at‐risk groups, including those with cardiovascular or respiratory diseases.^1^ At‐risk population also include vulnerable people such as residents of aged care facilities and other institutions and people who are at high risk of disease during influenza outbreaks. The attack rate of seasonal influenza varies with age and by type of influenza virus. The highest rate of influenza A/H3N2 strain is seen among children aged <5 years and in adults aged ≥65 years, whereas Influenza B disproportionately affects older children and young adults compared to influenza A.^2^, ^3^

Current evidence suggests that influenza infection is a precipitant for ischaemic events and that vaccination and prevention of infections may have an important role in reducing the ischaemic burden.^4^ From the Australian Bureau of Statistics, ischaemic heart disease (IHD) is the leading cause of death and responsible for 11% of all deaths in 2019.^5^ According to the Australian Institute of Health and Welfare (AIHW), cardiovascular disease (CVD) accounts for about 41,000 deaths as the underlying cause of death in Australia in 2018, equating to 26% of all deaths.^6^ Moreover, CVD was responsible for about 1.2 million hospitalisations in 2017–2018 and accounts for 11% of all hospitalisations in Australia. Hospitalisation rates of acute myocardial infarction, cardiovascular illness and all‐cause death increase during influenza season.^7^, ^8^, ^9^, ^10^ In a study, 12.4% of people admitted with AMI had undiagnosed, unrecognised influenza infection, compared to 6.7% of controls who did not have AMI, where it suggests a role of influenza.^11^ A study of over 80,000 influenza cases found that 12% had an acute cardiovascular event complicating their admission.^12^

Vaccination is the most effective public health intervention for prevention of influenza infection.^13^ In Australia, conventional and enhanced influenza vaccines are provided under the NIP. Quadrivalent influenza vaccine (QIV) was available for use from 2016–2017 in Australia, and high‐dose (HD) and adjuvanted TIVs were introduced for adults aged ≥65 years under the NIP in 2018, whilst other age groups received standard QIV. Increased efficacy of influenza vaccines has been shown to be associated with reduction of cardio‐respiratory illness and all‐cause hospitalisation in older adults.^14^, ^15^ Despite compelling evidence, the role of infection in ischaemic events is rarely counted in burden of disease estimates and in economic evaluations of the influenza vaccine. The aim of this study was to estimate the burden of respiratory and circulatory diseases attributable to influenza in adults aged 50–64 and ≥65 years in Australia.

## 2 METHODS

We used descriptive epidemiology and statistical modelling method to estimate the age‐specific rate of influenza‐attributable hospitalisations and deaths due to influenza/pneumonia, respiratory, circulatory and myocardial infarction (MI) in two age groups: 50–64 and ≥65 years, from July 2001 through December 2017. Our study aimed to highlight the gap in the vaccination programme between the two age groups to reduce influenza burden in Australia. We extracted surveillance data on weekly counts of laboratory‐confirmed influenza notifications in Australia between 2001 and 2018 from the National Notifiable Diseases Surveillance System (NNDSS), the Australian Government, Department of Health.^16^ Hospital admissions between 2001–2002 and 2017–2018 as well as deaths reported between 2001 and 2017 were requested under ICD‐10 principal diagnosis codes for influenza/pneumonia (J09‐J18), respiratory (J00–J99), circulatory (I00–I99) and MI (I21–I22). Hospitalisations and mortality data were obtained from the Australian Institute of Health and Welfare (AIHW)^17^ and the Australian Bureau of Statistics (ABS), respectively.^18^ Population data were sourced from the ABS.^19^ Rate per 100,000 population was calculated for 50–64 and ≥65 years.

### 2.1 Model

A semi‐parametric generalised‐additive model (GAM) was used to model the burden of influenza in 50–64 and ≥65 years.^20^, ^21^ Separate models were used for each age group and principal diagnosis category and estimated the age‐specific rate of influenza‐attributable hospitalisations and deaths in the study. Time series (weekly counts) of all‐age influenza notifications were entered into the model as predictor variables. Furthermore, an interaction term was used as value 0 or 1 between year and influenza notifications to include notifications as separate variables for each year. This at least allowed for changes in laboratory testing and reporting practices by healthcare providers over time during the study period. We adjusted for national holiday effects as reduced trend in hospital admissions was seen during holidays in the observed data. We applied separate indicator variables for each holiday (value 0 or 1) representing a week with a public holiday in it across the years. These variables provided varied effect across the models. We used a natural cubic smoothing spline of the consecutive week number to account for unmeasured non‐influenza‐attributable hospitalisations or deaths due to seasonal and other factors in the model. The benefit of using GAM is that as it allows the analysis of non‐linear association between the dependent and independent variables.^20^

The model equation was as follows:

$$Y=\beta_{0}+\sum_{\text{year}=\mathrm{Jul}\;2001}^{\mathrm{Jun}\;2018}\beta_{1,\text{year}}\left(\text{total influenza}\;\right)+\beta_{2}\left(\text{week before Xmas}\right)+\beta_{3}\left(\text{Xmas week}\right)+\beta_{4}\left(\mathrm{new}\;\text{year}’s\;\text{week}\right)+\beta_{5}\left(\mathrm{one}\;\text{week after}\;\mathrm{new}\;\text{year}\right)+\beta_{6}\left(\mathrm{two}\;\text{weeks after}\;\mathrm{new}\;\text{year}\right)+\beta_{7}\left(\text{three weeks after}\;\mathrm{new}\;\text{year}\right)+\beta_{8}\left(\text{week},\text{Australia}\;\mathrm{day}\right)+\beta_{9}\left(\mathrm{one}\;\text{week before Easter}\right)+\beta_{10}\left(\text{Easter week}\right)+\beta_{11}\left(\text{week},\text{ANZAC}\;\mathrm{Day}\right)+\beta_{12}\left(\text{time}\right)+\text{spline}\;\left(\text{time}\right)+\text{error}\;\left(\text{time}\right),$$

where *Y* represents the weekly hospitalisation rate per 100,000 population for a specific age group and disease category. β_0_ is the model intercept and β_1_ (year), β_2_, β_3_, β_4_, …, β_11_ represent the value of parameter estimates of the respective independent or indicator variable. β_12_ (time) is a linear term for week number to control for any long‐term (ecological) linear time trend in the hospitalisation rate, and spline (time) is a smoothing spline of the week number to control for unmeasured time varying factors.

Similarly, to estimate influenza‐attributable mortality for the period, 2001–2017, the modified version of the model was used separately for both age groups and four diagnostic categories. It was assumed that death may occur sometimes after exposure to the infection and the interval between the onset of symptoms and its associated death may occur from 1–40 days after the infection.^22^, ^23^ Hence, to allow the time delayed with mortality, influenza variable was lagged by 2 weeks in the mortality model, to reflect seasonal trend in the model outputs. Also, holiday variables were not included in the mortality model. The model equation for mortality is presented below.

$$Y=\beta_{0}+\sum_{\text{year}=\mathrm{Jan}\;2001}^{\mathrm{Dec}\;2017}\beta_{1,\text{year}}\left(\text{total influenza}\right)+\beta_{2}\left(\text{time}\right)+\text{spline}\;\left(\text{time}\right)+\text{error}\;\left(\text{time}\right).$$

As mentioned previously, *Y* represents weekly mortality rate per 100,000 population for a specific age group and disease category. β_0_ is the model intercept, β_1_ (year) is the parameter estimate of the respective independent variable, β_2_ (time) is a linear term for week number, and spline (time) represents smoothing spline of the week number in the model.

In addition, we split the years as pre‐ (July 2001 to December 2008) and post‐pandemic (January 2009 to June 2018) models for circulatory and MI due to variation in notification counts nationally since the pandemic year 2009.^2^, ^24^ Then, the models were run separately for estimated hospitalisation and mortality rates. We did not split years for influenza/pneumonia or respiratory model; instead, we applied a full model (all years, July 2001 to June 2018) here. The root mean‐squared error (RMSE) value was used to compare between the models, and lower RMSE value was considered a better fit of the model.^25^ For influenza/pneumonia or respiratory, all‐years model had better model fit compared to pre‐ and post‐pandemic models. For circulatory or MI, there were no apparent differences, and inconsistent RMSE values were obtained. We assumed that influenza attributed less to cardiovascular disease than respiratory illness; thus, instead of applying all years in the model, we trained the model using pre‐ and post‐pandemic years for circulatory and MI.

From the model, weekly age‐specific rate of hospitalisation or death per 100,000 population was estimated for each disease category, and annual total rates were calculated from the weekly estimates. For each disease category and age group, average seasonal estimate was calculated excluding the 2009 pandemic year in the analysis. A 95% conference interval (CI) was used to test the statistical significance in the study, and the analysis was conducted using SAS Enterprise Guide (SASEG) version 8.1 (SAS Institute Inc., Cary, NC, USA).

## 3 RESULTS

Over the study period, a total of approximately over 11 million (11,109,008) hospitalisations and nearly 1.2 million deaths (1,182,441) were extracted under four principal diagnosis category of influenza/pneumonia, respiratory, circulatory and MI among adults aged ≥50 years. Average annual rate of hospitalisation and death for both age groups by disease category are presented in Table 1. Mostly, older adults aged ≥65 years contributed a higher disease burden in general. Figures S1–S4 show the weekly observed rate of hospital admissions per 100,000 population in Australia. Seasonality was seen in both age groups for influenza/pneumonia and respiratory hospitalisations, whereas for circulatory and MI, seasonality was not evident in general except for MI in older adults.

**TABLE 1:** Observed, average yearly rate of hospitalisation and death per 100,000 population by principal diagnosis and age group, Australia, 2001–2017^a^

Principal diagnosis	Hospitalisation Rate/100,000		Mortality Rate/100,000	
	50–64 years	≥65 years	50–64 years	≥65 years
Influenza/pneumonia	287.6	1,390.9	3.6	90.8
Respiratory	1,301.2	4,528.9	22.6	379.1
Circulatory	3,022.7	9,741.0	93.9	1415.0
Myocardial infarction	373.5	1,039.9	32.4	455.9

^a^ July 2001 to December 2017 period, the 2009 pandemic year was excluded in the average calculation.

**FIGURE 1:**
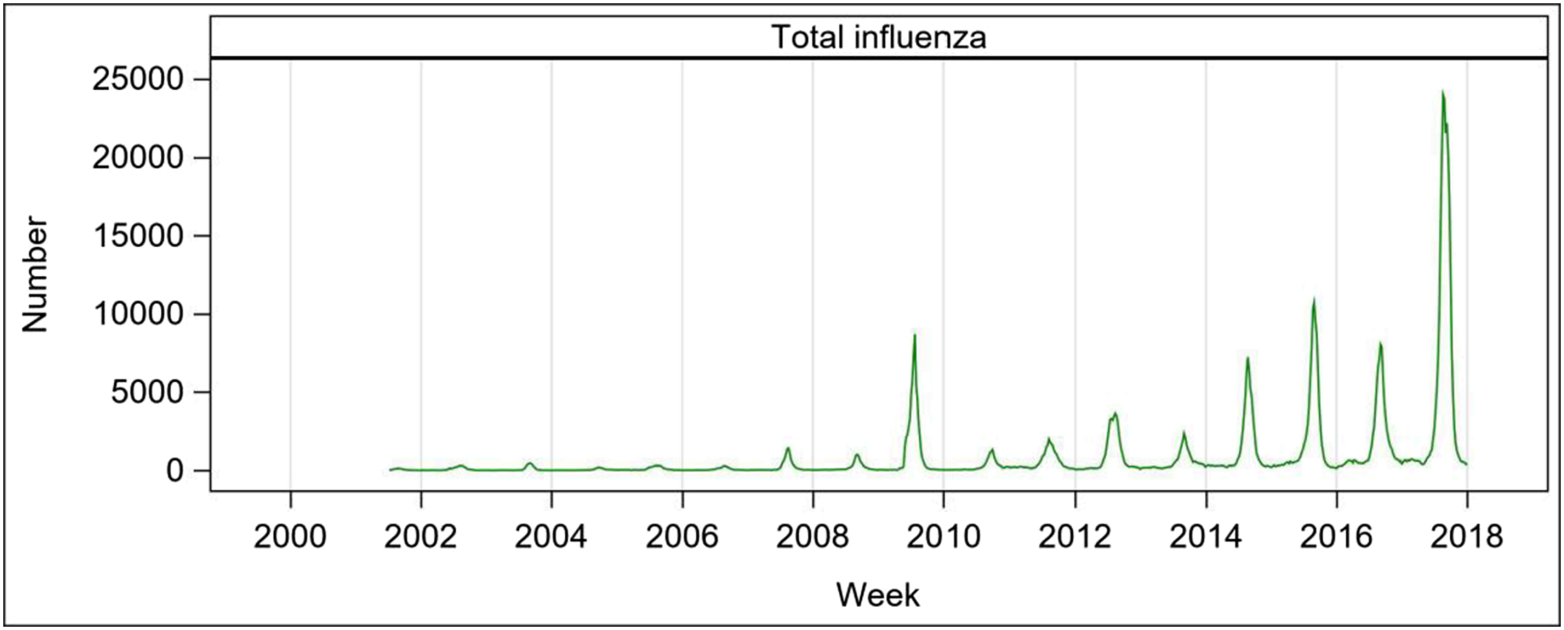
Laboratory‐confirmed influenza notifications in Australia, 2001–2017

Laboratory‐confirmed influenza notifications were extracted from the NNDSS for the study. The number of influenza notifications varied nationally from year to year over the study period (Figure 1). During the 17 seasons, the total reported laboratory‐confirmed cases of influenza were 728,663; the highest, 247,512, was recorded in 2017, whereas the lowest counts, 2,053, was observed in 2004. Distribution of influenza viruses across the studied years is shown in Figure S5.

### 3.1 Estimated hospitalisations attributable to influenza

**FIGURE 2:**
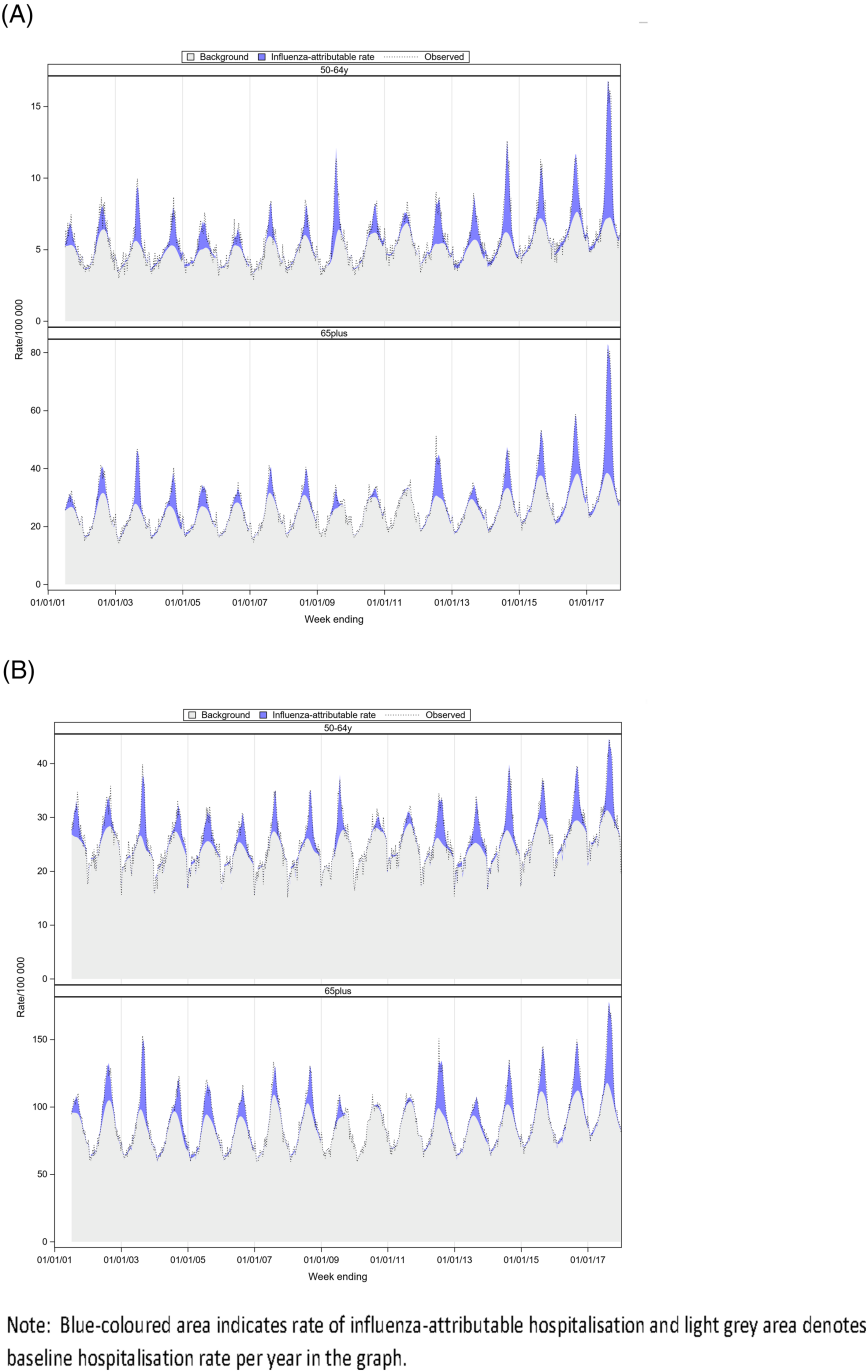
Estimated, observed and baseline rate of influenza‐attributable influenza/pneumonia and respiratory hospitalisation per 100,000 population, by year and age group, Australia, 2001–2017 (A) Influenza/pneumonia (B) Respiratory

The estimated hospitalisation rates varied across the seasons (Table S1). Compared to other years, 2017 season had the highest influenza‐attributable hospitalisation rate in both age groups and across respiratory diseases (Figure 2). Higher rates of influenza‐attributable respiratory admissions were observed in adults aged 65 years and over in years 2003, 2012 and 2017, where A/H3N2 strain circulated mostly during the season in Australia, and the estimated hospitalisation rates were 427.5 (95%CI: 403.3, 451.7), 468.3 (95%CI: 437.8, 498.8) and 638.0 (95%CI: 609.4, 666.7) per 100,000 population, respectively. In both age groups, the estimated hospitalisation rates seem very low in 2010 and 2011 compared to other years.

Estimated yearly rate of influenza‐attributable hospitalisation by age group and principal diagnoses of circulatory and MI are shown in the Figure S6 and Table S1. Influenza‐attributable MI hospitalisation rates were highest in 2005 in both age groups: 9.2 (95%CI: 3.9, 14.5) and 56.7 (95%CI: 44.0, 69.4) in adults aged 50–64 and 65 years and over, respectively. In older adults, significantly higher rates were observed in earlier years before 2009 (ranged from 14.2 to 56.7 per 100,000), followed by a rate of 16.0 (95%CI: 6.0, 26.1) in 2012 and 19.0 (95%CI: 9.6, 28.5) in 2017. Less association between influenza and MI hospitalisations was seen in recent years compared to pre‐pandemic period in adults aged 50–64 years, except 2017 where influenza‐attributable hospitalisation rate of 5.6 (95%CI: 1.3, 9.9) per 100,000 was estimated (Figure S6 and Table S1). Similarly, circulatory admissions did not show significant association with influenza in that age group except 2015 (estimated at 24.8 [95%CI: 2.9–46.8] per 100,000 people). In older adults, some years showed significant association between influenza and circulatory admissions, and higher hospitalisation rates were seen in 2002, population rate of 168.2 (95%CI: 104.2, 232.3), in 2005, 193.0 (95%CI: 123.0, 263.0) and in 2015, 131.7 (95%CI: 77.0, 186.4) per 100,000.

Table 2 shows the estimated average seasonal hospitalisation rate per 100,000 people attributable to influenza over the studied years. In adults aged ≥65 years, influenza attributed an average seasonal annual hospitalisation rate of 287.5 (95%CI: 279.8, 295.3) for respiratory and 123.6 (95%CI: 120.2, 126.9) for influenza/pneumonia. In adults aged 50–64 years, the corresponding rates were 78.9 (95%CI: 76.3, 81.4) and 32.3 (95%CI: 31.2, 33.3), respectively. For circulatory disease, seasonal average annual hospitalisation rate attributable to influenza was 57.2 (95%CI: 42.1, 72.3) for aged ≥65 years and 0.7 (95%CI: −5.2, 6.6) for adults aged 50–64 years per 100,000 population. For MI, the corresponding rates were 13.9 (95%CI: 11.2, 16.6) and 1.2 (95%CI: 0.1, 2.4), respectively.

**TABLE 2:** Estimated seasonal average annual influenza‐attributable hospitalisation rate per 100,000 population by principal diagnosis and age group, Australia, 2001–2017^a^

Principal diagnosis	Age group			
	50–64 years		≥65 years	
	Rate (95% CI)	Counts (95% CI)	Rate (95% CI)	Counts (95% CI)
Influenza/pneumonia	32.3 (31.2, 33.3)	1,287 (1,247, 1,326)	123.6 (120.2, 126.9)	3,975 (3,872, 4,077)
Respiratory	78.9 (76.3, 81.4)	3,071 (2,972, 3,170)	287.5 (279.8, 295.3)	8,869 (8,632, 9,106)
Circulatory	0.7 (−5.2, 6.6)	10 (−219, 240)	57.2 (42.1, 72.3)	1,650 (1,194, 2,107)
Myocardial infarction	1.2 (0.1, 2.4)	45 (0, 90)	13.9 (11.2, 16.6)	377 (297, 458)

Abbreviation: CI, confidence interval.

^a^ July 2001 to December 2017 period, the 2009 pandemic year was excluded in the average calculation, and both statistically significant and non‐significant estimates were included in the average.

### 3.2 Estimated mortality attributable to influenza

For elderly adults, statistically significant association was found between influenza and all four diseases; however, less mortality burden was seen in adults aged 50–64 years. For people aged ≥65 years, the estimated average annual mortality rates attributable to influenza were 37.7 (95%CI: 34.4, 41.0) for circulatory, 18.2 (95%CI: 16.9, 19.4) for respiratory, 12.8 (95%CI: 11.3, 14.3) for MI and 7.6 (95%CI: 7.0, 8.2) for influenza/pneumonia per 100,000 population (Table 3). The highest, approximately 1,100 circulatory deaths per year, was attributable to influenza in older adults. In adults 50–64 years, influenza attributed less mortality burden across all diseases and the lowest was observed for MI deaths, accounted for 12 deaths per annum (Table 3).

**TABLE 3:** Estimated seasonal average annual influenza‐attributable mortality rate per 100,000 population by principal diagnosis and age group, Australia, 2001–2017^a^

Principal diagnosis	Age group			
	50–64 years		≥65 years	
	Rate (95% CI)	Counts (95% CI)	Rate (95% CI)	Counts (95% CI)
Influenza/pneumonia	0.5 (0.4, 0.6)	21 (17, 25)	7.6 (7.0, 8.2)	244 (227, 262)
Respiratory	0.9 (0.7, 1.2)	37 (27, 46)	18.2 (16.9, 19.4)	564 (527, 602)
Circulatory	0.9 (0.4, 1.4)	34 (14, 54)	37.7 (34.4, 41.0)	1,080 (982, 1,178)
Myocardial infarction	0.3 (0.0, 0.6)	12 (1, 23)	12.8 (11.3, 14.3)	361 (316, 407)

Abbreviation: CI, confidence interval.

^a^ January 2001 to December 2017, the 2009 pandemic year was excluded in the average calculation, and both statistically significant and non‐significant estimates were included in the average.

Yearly estimated influenza‐attributable mortality rates are presented in Table S2. In adults aged ≥65 years, respiratory mortality rates were high in 2002, 2003, 2005, 2012, 2015, 2016 and 2017 in both influenza/pneumonia and respiratory deaths (Figure S7). Influenza‐attributable mortality rates were high in 2017: 47.2 (95%CI: 42.7, 51.8) for respiratory and 36.1 (95%CI: 34.0, 38.3) for influenza/pneumonia per 100,000 were found in older adults. In 2010 and 2011, estimated influenza/pneumonia mortality rates were lower than expected in adults 50–64 years, and these areas were seen as downward shaded areas in the graph shown in Figure S7. In 2017, influenza‐attributable mortality rates in adults 50–64 years were similar: 1.9 (95%CI: 1.5, 2.2) for influenza/pneumonia and 1.9 (95%CI: 1.0, 2.8) for respiratory per 100,000 population. Respiratory mortality rates were also increased in 2005, 2012, 2014 and 2016 in young adults attributable to influenza (Table S2).

**FIGURE 3:**
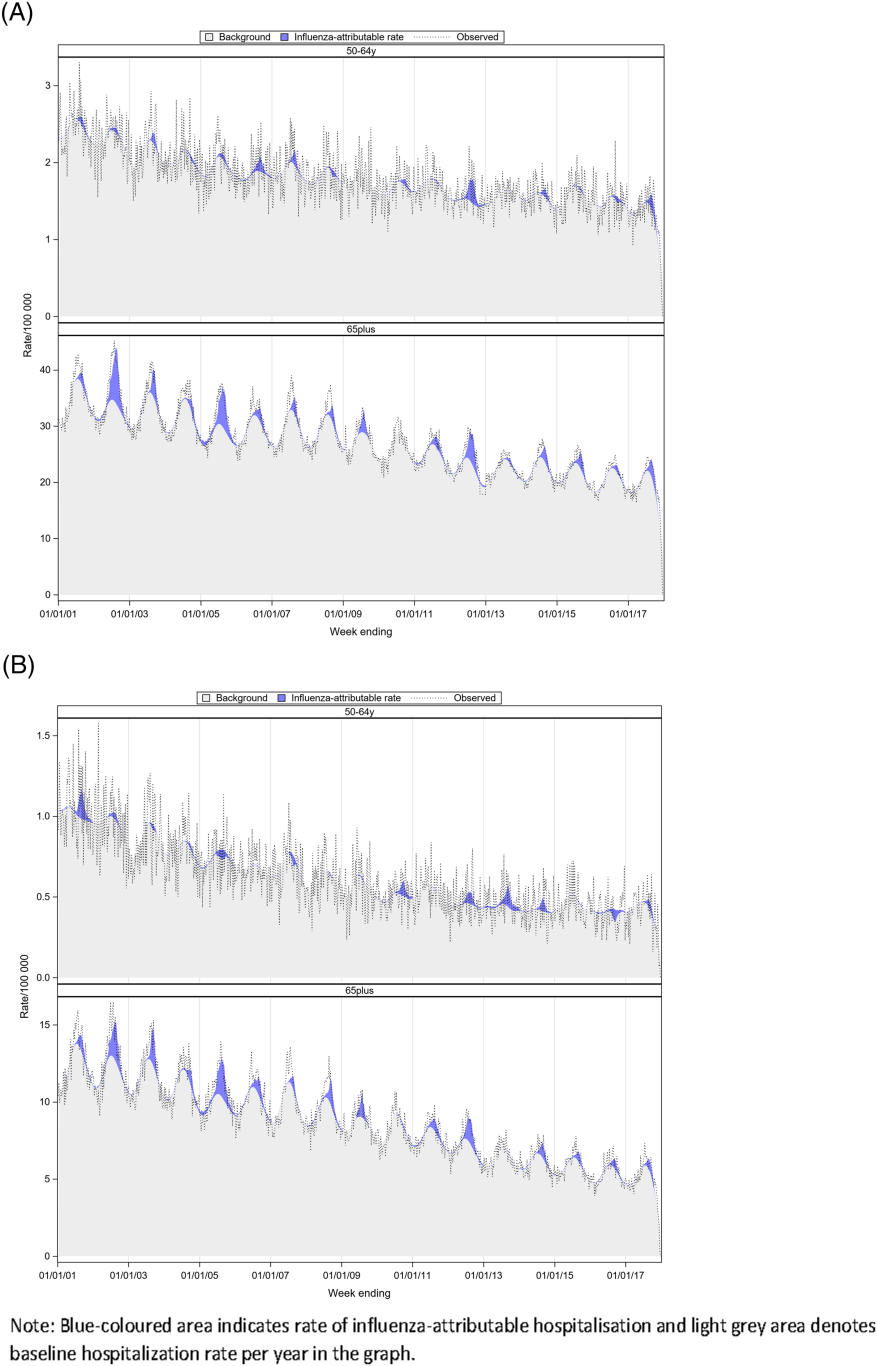
Estimated, observed and baseline rate of influenza‐attributable circulatory and MI deaths per 100,000 population, by year and age group, Australia, 2001–2017 (A) Circulatory (B) Myocardial infraction

In adults aged 50–64 years, influenza‐attributable MI and circulatory mortality rates were not statistically significant almost all years in the study except for circulatory, where influenza‐attributable mortality rate of 3.9 (95%CI: 1.9, 5.9) per 100,000 (Table S2) was found in 2012. However, in adults ≥65 years, statistically significant association between influenza and MI and circulatory deaths was found (Figure 3). The highest rates in adults ≥65 years were influenza‐attributable mortality rate of 31.1 (95%CI: 24.8, 37.4) and 37.5 (95%CI: 30.6, 44.4) for MI in 2002 and 2005, respectively, and the corresponding values were 122.8 (95%CI: 108.8, 136.7) and 105.5 (95%CI: 90.2, 120.8) for circulatory deaths. In post‐pandemic period, the 2012 season represented the highest mortality rate of 20.5 (95%CI: 14.8, 26.2) for MI and 69.2 (95%CI: 56.9, 81.5) for circulatory per 100,000 population in older adults in the study (Table S2). During a severe A/H3N2 season in 2017, the corresponding mortality estimates for MI and circulatory in older adults were 7.5 (95%CI: 2.1, 12.8) and 31.9 (95%CI: 20.4, 43.4) per 100,000, respectively.

## 4 DISCUSSION

The burden of influenza‐attributable complications was highest among adults ≥65 years old, but a considerable disease burden was also found in younger adults aged 50–64 years. Influenza was responsible for respiratory complications in both age groups, whereas less burden was seen in young adults 50–64 years old for cardiovascular complications. The estimated hospitalisation rates attributable to influenza varied by season and by age group. Across the years, 2017 season had the highest rate of influenza‐attributable respiratory complications in both age groups in the study. Australia experienced a severe A/H3N2 influenza season in 2017 with higher number of influenza notifications as well as increased reporting of influenza‐related hospitalisations and deaths recorded in the elderly during the season.^26^ Consistent to this, we found higher disease burden in our study in 2017. For people aged 65 years and over, our model estimated that influenza‐attributable respiratory hospitalisations was 11% of total respiratory admissions and influenza‐attributable respiratory deaths accounted for 13% of total notified respiratory mortality in 2017. The corresponding values for adults 50–64 years were 9% and 8% of total respiratory hospitalisations and respiratory deaths, respectively. Influenza‐attributable respiratory mortality rates were twice the value of their corresponding average annual rate for both age groups in 2017.

Compared to pre‐pandemic period, reporting of influenza notifications increased nationally after 2009.^27^, ^28^ This may be partly due to increased testing or reporting practices in the country after the pandemic.^24^ After 2017, respiratory hospitalisations were high during 2012 and 2016 influenza seasons in older adults, whilst the second highest influenza‐attributable respiratory morbidity was found in young adults in 2014. Influenza A/H3N2 virus predominated during 2017, 2012 and 2016, and some activity of influenza type B was also reported in 2016. It was documented that A/H3 virus disproportionately affected the older adults, whereas A/H1N1pdm09 strain affected more towards the younger age.^26^, ^29^ Consistently, our study estimated higher respiratory morbidity and mortality in 2014 in those adults aged 50–64 years.

Our study estimated an average annual respiratory hospitalisation rate of 288 per 100,000 population in adults aged ≥65 years. This is comparable to previous Australian study by Newall et al.,^30^ for the period from 1997/98 to 2004/05 (Tables S3). In addition, current hospitalisation estimates are similar to the results of recently published study, where influenza‐attributable hospitalisations were estimated for influenza/pneumonia and respiratory diseases from 2001 to 2013.^21^ Both studies found that on average, per year influenza accounted for 6,798 to 8,869 respiratory admissions in older adults aged ≥65 years. For respiratory mortality, our mortality estimates were slightly higher than previous study by Muscatello et al.,^20^ from Australia conducted for the period 2003–2009. Our estimates for aged ≥65 years are lower than the estimated rates of respiratory hospitalisation and mortality from Hong Kong (847.2 and 48.7) for 1998–2013,^31^ and pneumonia and influenza hospitalisation rate of 338.0 per 100,000 from Singapore, 2010–2017.^32^ Also, our estimated influenza/pneumonia hospitalisation rates are comparable to influenza hospitalisation rates reported from the United States for both age groups: 50–64 and >65 years.^33^ However, due to differences in study period, inclusion of varied disease category and diverse age group, and type of statistical modelling methods applied, the outcome results are varied across the studies.^20^, ^21^, ^30^

In addition to respiratory complications, we found that on average, influenza accounted for 2.8% and 1% of total MI deaths annually in adults aged ≥65 years and 50–64 years, respectively. For circulatory and MI, 2002 and 2005 showed higher rates of influenza‐attributable hospitalisations and deaths among elderly adults. These seasons have been reported with moderate seasonal activity in Australia; however, both influenza A and B outbreaks were also reported during the two seasons.^34^, ^35^ Antigenic drift of A/H3 strain and report of type B lineage mismatch with the vaccine strain were recorded during 2005,^2^, ^34^, ^35^ and A/H3 strain was the dominant circulating strain reported in 2002. However, it should be noted that influenza surveillance programme was initiated in Australia in 2001 and was at its early stage of development; thus, notifications may had been underreported nationally in 2002.

Influenza triggers cardiovascular‐related complications, and many studies documented influenza‐attributable cardiovascular events.^12^, ^36^, ^37^, ^38^, ^39^ In a study, adults who were admitted with influenza, 6.2% and 5.7%, had heart failure and ischaemic heart disease, respectively.^12^ Acute association between influenza and sudden onset of cardiovascular events or complications of pre‐existing comorbidities in older adults leads to sudden deaths and increased influenza‐related circulatory mortality in the season.^7^, ^40^ The association between influenza and MI is significant. Our study estimated that influenza‐attributable MI deaths accounted for 33% of circulatory deaths due to influenza and influenza‐attributable MI hospitalisations were 23% of circulatory hospitalisations attributable to influenza in adults aged ≥65 years. Though the association between influenza and cardiac events in adults aged 50–64 years appears less evident compared to older age group, MI mortality in young adults attributed 35% of all influenza‐related circulatory deaths in the study. We found that there was reduction in circulatory mortality in recent years in general. This is comparable to mortality trend reported nationally from the ABS, where a decline in mortality was reported in Australia for IHD and cerebrovascular diseases for the last 10 years (2010–2019).^5^

It is evident that influenza vaccine offers protective effect on influenza‐related cardiac events.^37^, ^41^ A meta‐analysis of five randomised controlled trials demonstrated that influenza vaccine could reduce cardiovascular events by 36% among patients who are at high‐risk of the disease.^42^ A meta‐analysis of observational studies showed that vaccine is effective in preventing AMI with an estimated VE of 29%.^36^ In Australia, influenza vaccine coverage remains constant at 70–75% in adults ≥65 years; however, the coverage in adults <65 years and the targeted at‐risk population remains suboptimal and under 50%.^43^, ^44^ From our estimates, influenza accounted for a considerable disease burden in adults 50–64 years. Due to lower vaccine coverage, thus, prevention is vital in young adults, especially for individuals who are at increased risk of infection and its complications. A recent study on economic benefits of influenza vaccine showed that the extension of current vaccination programme offers an extra cost savings and an additional net cost saving of 8 million by the government if the vaccination programme extended for adults aged 50–64 years under the NIP.^45^ Consequently, additional effect of influenza vaccine in preventing CVD events in addition to respiratory complications in young adults could contribute large economic and health benefits in the community.

This study has some limitations. First, in the study, weekly counts of total influenza were applied in the model; thus, type‐specific disease burden cannot be estimated in these age groups. Second, the seasonality of influenza infection may coincide with seasonality of respiratory syncytial viruses (RSV), and our study could not be able to adjust presence of RSV in the model; thus, study findings may have been confounded by RSV. Finally, in the study, we used national data on weekly counts of all‐age influenza infection for individual year in the model, to account for variation in testing or reporting practices by general practitioners as well as variation across states and territories in Australia over time. Strengths of the study include the use of weekly counts of total influenza infection as a proxy measure or covariate in the model, thus reflecting the incidence of infection in the population to predict better disease estimates across the studied years. Also, 17 years of influenza notifications data in the study could provide a stronger statistical power in model outputs compared to a short‐term data analysis.

In conclusion, our study demonstrates that influenza attributed to increased respiratory morbidity in both age groups: 50–64 and ≥65 years and high circulatory mortality among the older population. Influenza and its associated complications can be prevented by seasonal vaccines. Improved vaccine uptake in the recommended age group, as well as in adults 50–64 years of age including the high‐risk population, might be the best available intervention to reduce cardiorespiratory disease burden associated with influenza.

## AUTHOR CONTRIBUTIONS

**Aye Moa:** Data curation; formal analysis; methodology. **Robert Menzies:** Formal analysis; methodology. **J. Kevin Yin:** Conceptualization; formal analysis; methodology. **C. Raina MacIntyre:** Conceptualization; formal analysis; methodology.

## Supporting information


**Table S1.** Estimated annual influenza‐attributable hospitalisation rate by principal diagnosis and year, 50–64 and 65 years and over, Australia, 2001–2017.
**Table S2.** Estimated annual influenza‐attributable mortality rate by principal diagnosis and year, 50–64 and 65 years and over, Australia, 2001–2017.
**Table S3.** Comparison of estimated influenza‐attributable hospitalisation and mortality between studies.
**Figure S1:** Observed rate of hospitalisation with principal diagnosis of respiratory disease.
**Figure S2:** Observed rate of hospitalisation with principal diagnosis of influenza/pneumonia.
**Figure S3:** Observed rate of hospitalisation with principal diagnosis of circulatory disease.
**Figure S4:** Observed rate of hospitalisation with principal diagnosis of myocardial infraction
**Figure S5.** Laboratory‐confirmed influenza notifications by type, subtype and year, Australia, 2001–2017.
**Figure S6:** Estimated, observed and baseline annual hospitalisation rate* of influenza‐attributable circulatory and myocardial infraction, by year and age group, Australia, 2001–2017
**Figure S7:** Estimated, observed and baseline annual rate* of influenza‐attributable influenza/pneumonia and respiratory death, by year and age group, Australia, 2001–2017.

## Data Availability

Data are available from the Australian Institute of Health and Welfare and the Australian Bureau of Statistics, Institutional Data Access for researchers who meet the criteria for access to confidential data. Surveillance data is publicly available from the National Notifiable Diseases Surveillance System, Department of Health, Australian Government, URL: National Notifiable Diseases Surveillance System (NNDSS) (health.gov.au)
