# Supplementary material for: Modelling the influenza disease burden in people aged 50–64 and ≥65 years in Australia
Source: Influenza Other Respir Viruses. 2021 Sep 29;16(1):132–41. doi: 10.1111/irv.12902 (PMC8692809; doi:10.1111/irv.12902)
Supplement: Supplementary file 1 — Table S1. Estimated annual influenza‐attributable hospitalisation rate by principal diagnosis and year, 50–64 and 65 years and over, Australia, 2001–2017. Table S2. Estimated annual influenza‐attributable mortality rate by principal diagnosis and year, 50–64 and 65 years and over, Australia, 2001–2017. Table S3. Comparison of estimated influenza‐attributable hospitalisation and mortality between studies. Figure S1: Observed rate of hospitalisation with principal diagnosis of respiratory disease. Figure S2: Observed rate of hospitalisation with principal diagnosis of influenza/pneumonia. Figure S3: Observed rate of hospitalisation with principal diagnosis of circulatory disease. Figure S4: Observed rate of hospitalisation with principal diagnosis of myocardial infraction Figure S5. Laboratory‐confirmed influenza notifications by type, subtype and year, Australia, 2001–2017. Figure S6: Estimated, observed and baseline annual hospitalisation rate* of influenza‐attributable circulatory and myocardial infraction, by year and age group, Australia, 2001–2017 Figure S7: Estimated, observed and baseline annual rate* of influenza‐attributable influenza/pneumonia and respiratory death, by year and age group, Australia, 2001–2017. [file IRV-16-132-s001.pdf]

Supplementary

Table S1. Estimated annual influenza-attributable hospitalisation rate by principal diagnosis and year, 50-64 and 65 years and over, Australia, 2001-2017.

a. Influenza/pneumonia and respiratory

		Hospitalisation rate* – attributable to influenza (95%CI)	
Principal diagnosis	Year	Age group	
		50-64 years	≥65 years
Influenza/ pneumonia	2001 (Jul-Dec)	**14.6 (10.9, 18.2)**	**41.9 (29.8, 53.9)**
	2002	**20.0 (15.7, 24.2)**	**110.3 (96.3, 124.2)**
	2003	**29.7 (26.5, 32.9)**	**145.7 (135.2, 156.1)**
	2004	**29.3 (25.0, 33.6)**	**134.9 (120.7, 149.0)**
	2005	**27.7 (23.1, 32.3)**	**105.7 (90.4, 120.9)**
	2006	**13.6 (9.2, 18.0)**	**54.9 (40.4, 69.4)**
	2007	**17.7 (14.4, 21.1)**	**67.1 (56.1, 78.0)**
	2008	**19.3 (15.5, 23.1)**	**86.2 (73.7, 98.8)**
	2009	**42.0 (38.8, 45.2)**	**48.7 (38.2, 59.3)**
	2010	**20.7 (16.7, 24.8)**	**31.1 (17.7, 44.5)**
	2011	**12.0 (7.3, 16.8)**	-5.9 (-21.6, 9.8)
	2012	**38.3 (34.3, 42.2)**	**179.6 (166.4, 192.7)**
	2013	**36.3 (31.5, 41.1)**	**67.5 (51.7, 83.2)**
	2014	**59.0 (55.1, 62.8)**	**130.1 (117.5, 142.8)**
	2015	**33.4 (29.6, 37.1)**	**142.0 (129.6, 154.5)**
	2016	**45.7 (41.3, 50.1)**	**227.0 (212.6, 241.4)**
	2017	**98.9 (95.2, 102.6)**	**459.1 (446.7, 471.4)**
Respiratory	2001 (Jul-Dec)	**61.3 (52.1, 70.4)**	**116.8 (88.8, 144.7)**
	2002	**64.4 (53.8, 75.0)**	**332.1 (299.8, 364.4)**
	2003	**94.2 (86.3, 102.1)**	**427.5 (403.3, 451.7)**
	2004	**60.0 (49.3, 70.8)**	**319.6 (286.9, 352.4)**
	2005	**78.8 (67.3, 90.4)**	**342.9 (307.6, 378.2)**
	2006	**66.0 (55.0, 77.1)**	**241.2 (207.6, 274.8)**
	2007	**57.4 (49.0, 65.7)**	**161.3 (135.8, 186.7)**
	2008	**87.2 (77.6, 96.7)**	**278.9 (249.9, 308.0)**
	2009	**75.3 (67.3, 83.3)**	**111.6 (87.1, 136.0)**
	2010	**24.1 (13.9, 34.3)**	20.5 (-10.6, 51.6)
	2011	**33.9 (22.0, 45.8)**	**53.5 (17.2, 89.8)**
	2012	**100.0 (90.0, 110.0)**	**468.3 (437.8, 498.8)**
	2013	**100.3 (88.4, 112.3)**	**197.2 (160.8, 233.6)**
	2014	**116.0 (106.4, 125.7)**	**300.2 (270.8, 329.6)**
	2015	**67.7 (58.2, 77.1)**	**306.0 (277.2, 334.9)**
	2016	**111.3 (100.4, 122.3)**	**396.6 (363.2, 429.9)**
	2017	**139.3 (129.9, 148.7)**	**638.0 (609.4, 666.7)**

* Rate per 100,000 population.
Statistically significant years are presented in bold font.

b. Myocardial infarction and circulatory

		Hospitalisation rate* – attributable to influenza (95%CI)	
Principal diagnosis	Year	Age group	
		50-64 years	≥65 years
Myocardial infarction	2001 (Jul-Dec)	*-5.9 (-10.1, -1.7)*	**19.1 (9.0, 29.1)**
	2002	2.8 (-2.0, 7.7)	**28.9 (17.2, 40.5)**
	2003	3.1 (-0.5, 6.7)	**27.0 (18.2, 35.7)**
	2004	0.4 (-4.5, 5.3)	**18.9 (7.1, 30.7)**
	2005	**9.2 (3.9, 14.5)**	**56.7 (44.0, 69.4)**
	2006	**6.3 (1.3, 11.4)**	**14.6 (2.5, 26.8)**
	2007	2.2 (-1.7, 6.0)	**15.0 (5.9, 24.2)**
	2008	**6.2 (1.9, 10.6)**	**14.2 (3.7, 24.6)**
	2009	3.3 (-0.4, 6.9)	**12.9 (4.8, 20.9)**
	2010	*-6.2 (-10.9, -1.6)*	0.8 (-9.4, 11.0)
	2011	-1.9 (-7.3, 3.5)	-4.4 (-16.3, 7.5)
	2012	2.8 (-1.7, 7.4)	**16.0 (6.0, 26.1)**
	2013	-0.3 (-5.7, 5.1)	-7.4 (-19.4, 4.6)
	2014	-0.2 (-4.6, 4.2)	-3.5 (-13.2, 6.2)
	2015	*-5.4 (-9.7, -1.1)*	6.8 (-2.7, 16.3)
	2016	1.1 (-3.9, 6.1)	0.1 (-10.9, 11.1)
	2017	**5.6 (1.3, 9.9)**	**19.0 (9.6, 28.5)**
Circulatory	2001 (Jul-Dec)	3.1 (-18.2, 24.3)	39.0 (-16.4, 94.3)
	2002	17.3 (-7.3, 41.9)	**168.2 (104.2, 232.3)**
	2003	-15.9 (-34.4, 2.5)	24.1 (-23.9, 72.2)
	2004	9.2 (-15.8, 34.1)	34.1 (-30.9, 99.1)
	2005	0.2 (-26.7, 27.1)	**193.0 (123.0, 263.0)**
	2006	26.0 (0.3, 51.6)	**78.9 (12.1, 145.7)**
	2007	-15.7 (-35.1, 3.7)	14.6 (-35.9, 65.1)
	2008	20.4 (-1.7, 42.6)	**64.4 (6.8, 122.1)**
	2009	-5.0 (-23.6, 13.6)	**48.5 (2.2, 94.8)**
	2010	7.8 (-15.8, 31.4)	6.2 (-52.7, 65.0)
	2011	-6.4 (-34.1, 21.2)	10.7 (-58.1, 79.5)
	2012	1.2 (-22.0, 24.4)	48.8 (-9.1, 106.6)
	2013	*-44.1 (-71.9, -16.4)*	-8.7 (-77.8, 60.4)
	2014	0.6 (-21.8, 23.0)	47.7 (-8.1, 103.5)
	2015	**24.8 (2.9, 46.8)**	**131.7 (77.0, 186.4)**
	2016	-3.7 (-29.1, 21.6)	**65.4 (2.2, 128.7)**
	2017	-13.4 (-35.2, 8.4)	-2.9 (-57.2, 51.4)

* Rate per 100,000 population.
Statistically significant years are presented in bold font.

Table S2. Estimated annual influenza-attributable mortality rate by principal diagnosis and year, 50-64 and 65 years and over, Australia, 2001-2017.

a. Influenza/pneumonia and respiratory

		Death rate* – attributable to influenza (95%CI)	
Principal cause of death	Year	Age group	
		50-64 years	≥65 years
Influenza/ pneumonia	2001	-0.1 (-0.5, 0.3)	2.2 (0.0, 4.5)
	2002	-0.6 (-1.0, -0.2)	**13.3 (10.9, 15.7)**
	2003	0.3 (0.0, 0.6)	**11.7 (9.9, 13.5)**
	2004	0.2 (-0.2, 0.6)	2.3 (-0.2, 4.7)
	2005	0.6 (0.2, 1.1)	**13.1 (10.5, 15.7)**
	2006	0.2 (-0.3, 0.6)	**4.6 (2.0, 7.1)**
	2007	-0.2 (-0.5, 0.2)	2.7 (0.8, 4.6)
	2008	0.2 (-0.2, 0.6)	0.6 (-1.6, 2.8)
	2009	0.2 (-0.2, 0.5)	1.9 (0.0, 3.7)
	2010	1.2 (0.8, 1.6)	-0.1 (-2.5, 2.2)
	2011	1.6 (1.1, 2.1)	-1.1 (-3.9, 1.6)
	2012	0.5 (0.1, 0.9)	**10.3 (8.0, 12.6)**
	2013	0.4 (-0.1, 0.9)	-1.5 (-4.2, 1.3)
	2014	0.8 (0.4, 1.2)	**6.4 (4.2, 8.6)**
	2015	0.1 (-0.3, 0.5)	**10.4 (8.3, 12.6)**
	2016	0.9 (0.5, 1.3)	**11.0 (8.5, 13.5)**
	2017	**1.9 (1.5, 2.2)**	**36.1 (34.0, 38.3)**
Respiratory	2001	0.2 (-0.8, 1.1)	**9.5 (4.7, 14.3)**
	2002	0.1 (-0.9, 1.1)	**34.4 (29.3, 39.5)**
	2003	1.2 (0.5, 2.0)	**27.0 (23.1, 30.8)**
	2004	-0.3 (-1.3, 0.7)	**13.2 (8.1, 18.3)**
	2005	**2.6 (1.5, 3.7)**	**23.9 (18.3, 29.5)**
	2006	-0.3 (-1.4, 0.7)	**14.1 (8.8, 19.5)**
	2007	0.1 (-0.7, 0.9)	**4.1 (0.1, 8.1)**
	2008	0.2 (-0.7, 1.1)	**5.8 (1.2, 10.4)**
	2009	1.1 (0.4, 1.9)	**11.4 (7.6, 15.3)**
	2010	0.6 (-0.3, 1.6)	4.5 (-0.4, 9.3)
	2011	0.7 (-0.5, 1.8)	-1.9 (-7.7, 3.9)
	2012	**2.3 (1.4, 3.3)**	**30.1 (25.2, 34.9)**
	2013	-0.2 (-1.3, 1.0)	**11.4 (5.6, 17.2)**
	2014	**3.0 (2.1, 3.9)**	**21.3 (16.6, 25.9)**
	2015	-0.2 (-1.1, 0.7)	**22.1 (17.5, 26.7)**
	2016	**2.6 (1.6, 3.6)**	**24.4 (19.1, 29.7)**
	2017	**1.9 (1.0, 2.8)**	**47.2 (42.7, 51.8)**

* Rate per 100,000 population.
Statistically significant years are presented in bold font.

b. Myocardial infarction and circulatory

		Death rate* – attributable to influenza (95%CI)	
Principal cause of death	Year	Age group	
		50-64 years	≥65 years
Myocardial infarction	2001	1.9 (0.8, 3.0)	**10.8 (4.9, 16.8)**
	2002	0.5 (-0.7, 1.7)	**31.1 (24.8, 37.4)**
	2003	-0.2 (-1.2, 0.7)	**18.1 (13.4, 22.9)**
	2004	0.3 (-0.9, 1.6)	**10.5 (4.1, 16.8)**
	2005	-0.9 (-2.2, 0.4)	**37.5 (30.6, 44.4)**
	2006	0.0 (-1.2, 1.3)	**14.2 (7.6, 20.8)**
	2007	-0.5 (-1.4, 0.5)	**6.6 (1.6, 11.6)**
	2008	-0.1 (-1.2, 1.0)	**15.6 (9.9, 21.3)**
	2009	0.1 (-0.8, 1.0)	**11.0 (6.4, 15.5)**
	2010	1.0 (-0.2, 2.1)	-2.0 (-7.7, 3.8)
	2011	0.0 (-1.3, 1.4)	**11.3 (4.5, 18.1)**
	2012	0.9 (-0.2, 2.1)	**20.5 (14.8, 26.2)**
	2013	1.7 (0.3, 3.0)	0.5 (-6.3, 7.2)
	2014	0.8 (-0.4, 1.9)	**9.3 (3.8, 14.9)**
	2015	-0.1 (-1.2, 1.0)	**6.1 (0.7, 11.5)**
	2016	-0.9 (-2.1, 0.4)	**6.8 (0.6, 12.9)**
	2017	0.5 (-0.6, 1.6)	**7.5 (2.1, 12.8)**
Circulatory	2001	1.1 (-0.9, 3.1)	**22.3 (9.2, 35.5)**
	2002	0.7 (-1.4, 2.8)	**122.8 (108.8, 136.7)**
	2003	1.1 (-0.5, 2.7)	**39.2 (28.6, 49.7)**
	2004	-0.6 (-2.7, 1.5)	**23.6 (9.5, 37.7)**
	2005	1.0 (-1.3, 3.4)	**105.5 (90.2, 120.8)**
	2006	2.3 (0.1, 4.6)	**30.1 (15.4, 44.8)**
	2007	1.5 (-0.2, 3.2)	**23.1 (12.1, 34.2)**
	2008	-0.8 (-2.7, 1.1)	**26.8 (14.2, 39.5)**
	2009	0.0 (-1.6, 1.6)	**29.0 (19.2, 38.8)**
	2010	0.9 (-1.1, 2.9)	0.7 (-11.7, 13)
	2011	0.3 (-2.1, 2.7)	**28.7 (14.1, 43.4)**
	2012	**3.9 (1.9, 5.9)**	**69.2 (56.9, 81.5)**
	2013	0.1 (-2.3, 2.4)	10.8 (-3.8, 25.4)
	2014	1.0 (-0.9, 3.0)	**26.9 (15.1, 38.8)**
	2015	0.3 (-1.6, 2.2)	**27.7 (16.1, 39.4)**
	2016	-0.8 (-2.9, 1.4)	13.9 (0.6, 27.2)
	2017	2.1 (0.2, 3.9)	**31.9 (20.4, 43.4)**

* Rate per 100,000 population.
Statistically significant years are presented in bold font.

Table S3. Comparison of estimated influenza-attributable hospitalisation and mortality rate between two Australian studies.

a. Influenza-attributable hospitalisation rate per 100,000 population

	**Age (50-64 years)**			
	IP*	Respiratory	MI***	Circulatory
This study (Jun 2001-2017)	32.3 (31.2, 33.3)	78.9 (76.3, 81.4)	1.2 (0.1, 2.4)	0.7 (-5.2, 6.6)
Newall et al.[1] (Jul1998-Jun2005)	33.3 (23.2, 43.4)	57.6 ** (32.5, 82.8)	-	-1.5 (-58.8, 55.8)
	**Age (≥65 years)**			
This study	123.6 (120.2, 126.9)	287.5 (279.8, 295.3)	13.9 (11.2, 16.6)	57.2 (42.1, 72.3)
Newall et al.[1]	157.4 (108.4, 206.5)	282.0 ** (183.7, 380.3)	-	39.6 (-114.9, 194.1)

IP (J09-J18); Respiratory (J00-J99); MI (I21-I22); Circulatory (I00-I99)
*IP, Influenza/pneumonia; ** Other respiratory; ***MI, Myocardial infraction

b. Influenza-attributable mortality rate per 100,000 population

	**Age (50-64 years)**			
	IP*	Respiratory	MI***	Circulatory
This study (2001-2017)	0.5 (0.4, 0.6)	0.9 (0.7, 1.2)	0.3 (0.0, 0.6)	0.9 (0.4, 1.4)
Newall et al.[1] (Jul1998-Jun2005)	0.6 (0.3 to 1.0)	2.1 ** (1.2 to 3.0)	-	1.4 (−0.6 to 3.4)
	**Age (≥65 years)**			
This study	7.6 (7.0, 8.2)	18.2 (16.9, 19.4)	12.8 (11.3, 14.3)	37.7 (34.4, 41.0)
Newall et al.[1]	17.6 (12.9 to 22.3)	24.4 ** (18.8 to 30.0)	-	49.3 (27.0 to 71.6)

IP (J09-J18); Respiratory (J00-J99); MI (I21-I22); Circulatory (I00-I99).
*IP, Influenza/pneumonia; ** Other respiratory; ***MI, Myocardial infraction

Ref:

1. Newall AT, Wood JG, MacIntyre CR. Influenza-related hospitalisation and death in Australians aged 50 years and older. Vaccine. 2008;26(17):2135-2141.

Figures:

Figure S1: Observed rate of hospitalisation with principal diagnosis of respiratory disease.

a. ≥65 years

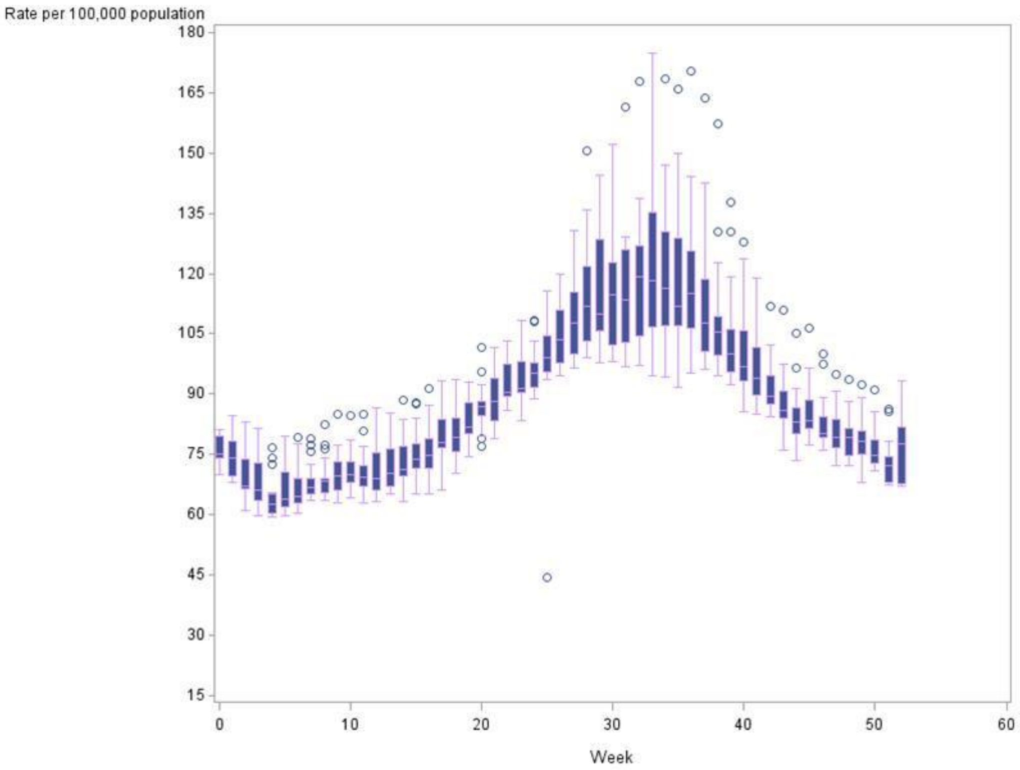

b. 50-64 years

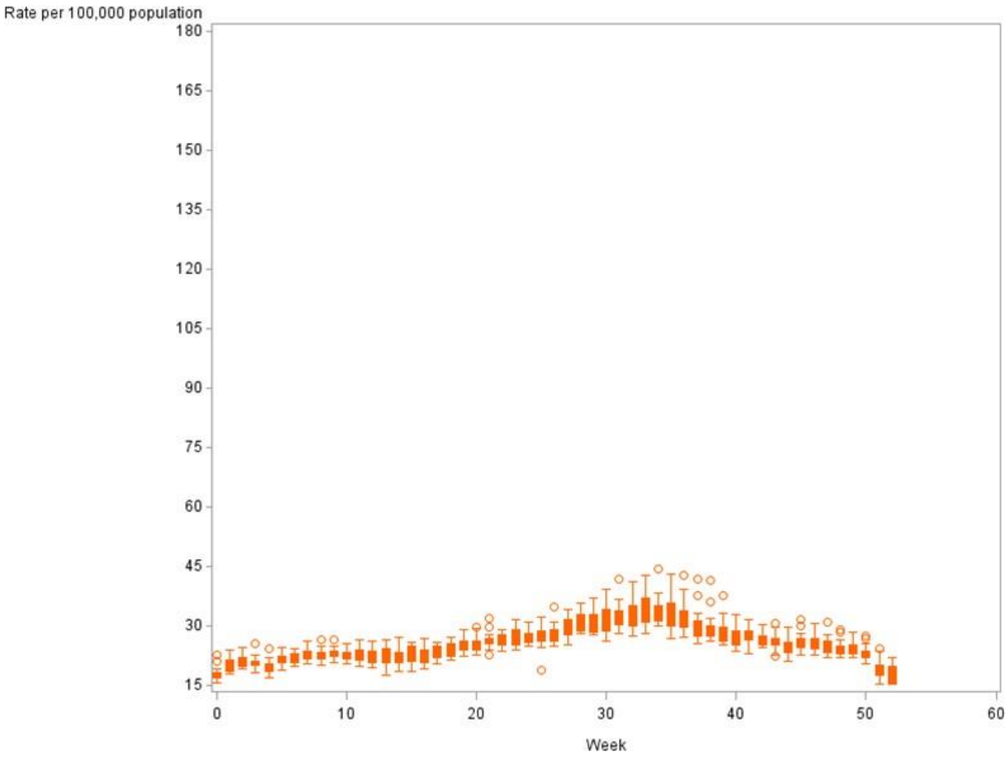

Figure S2: Observed rate of hospitalisation with principal diagnosis of influenza/pneumonia.

a. ≥65 years

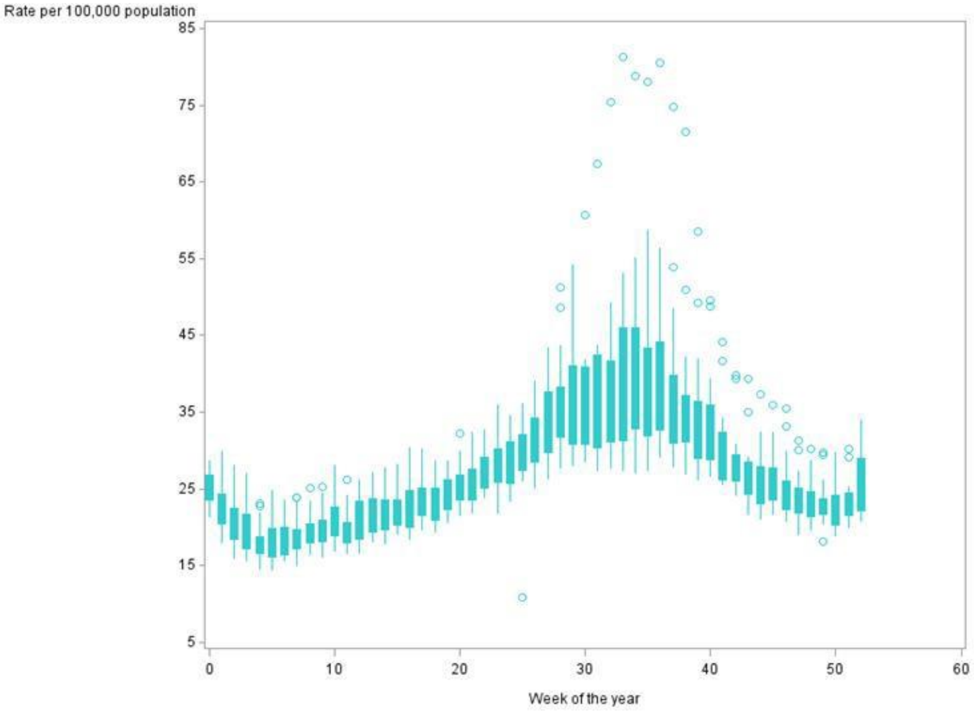

b. 50-64 years

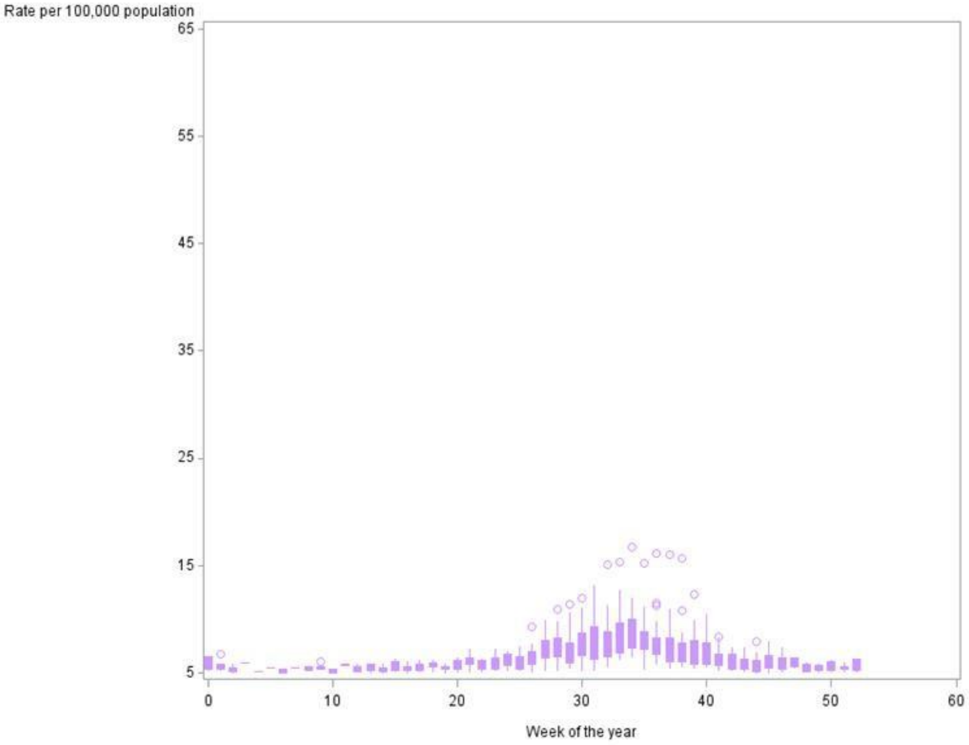

Figure S3: Observed rate of hospitalisation with principal diagnosis of circulatory disease.

a. ≥65 years

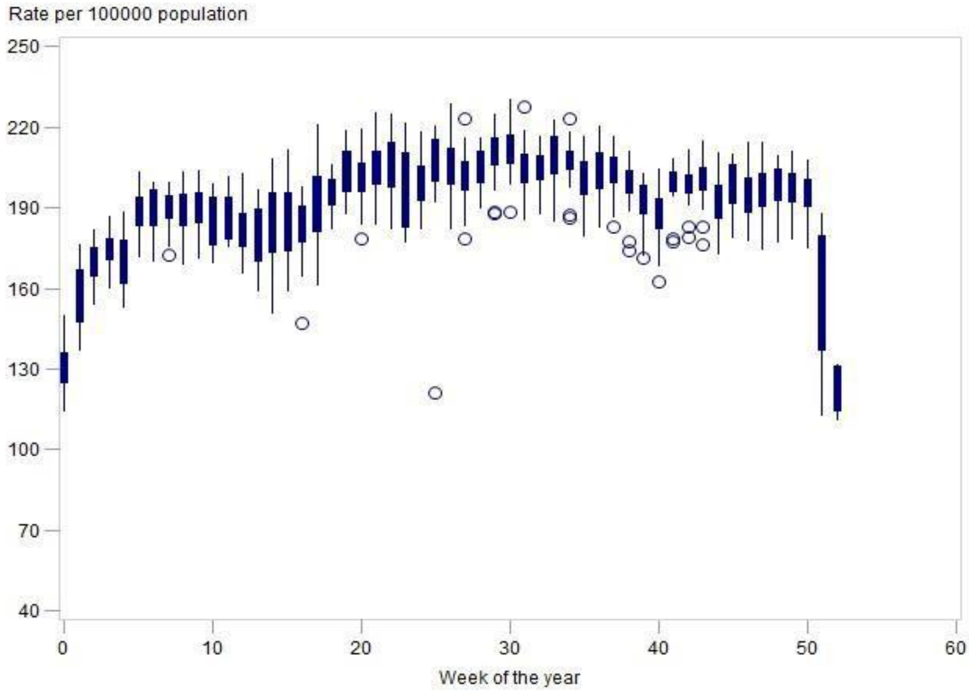

b. 50-64 years

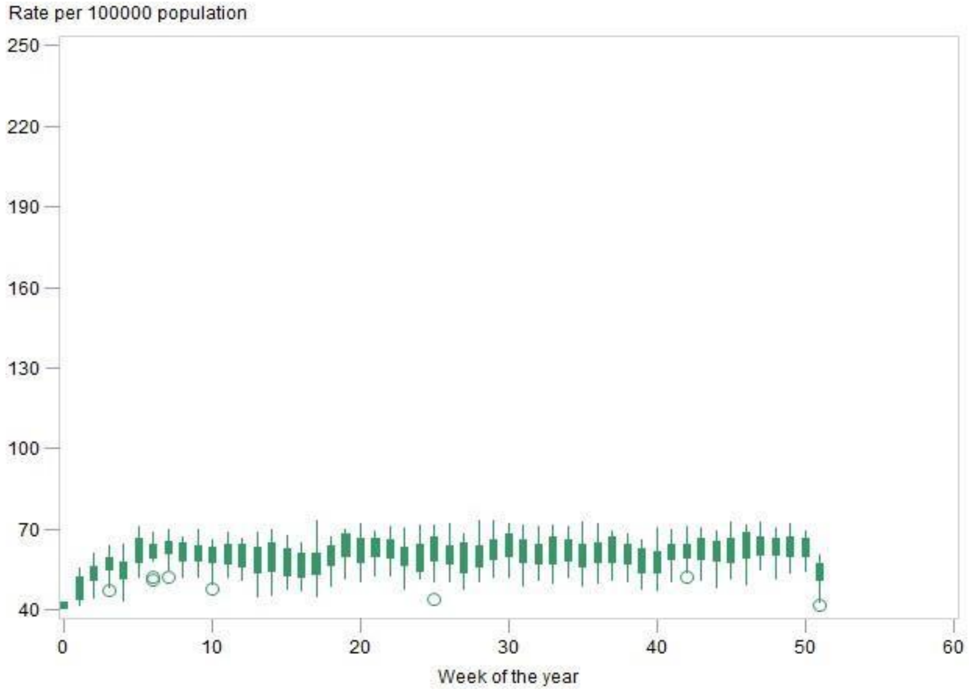

Figure S4: Observed rate of hospitalisation with principal diagnosis of myocardial infraction

a. ≥65 years

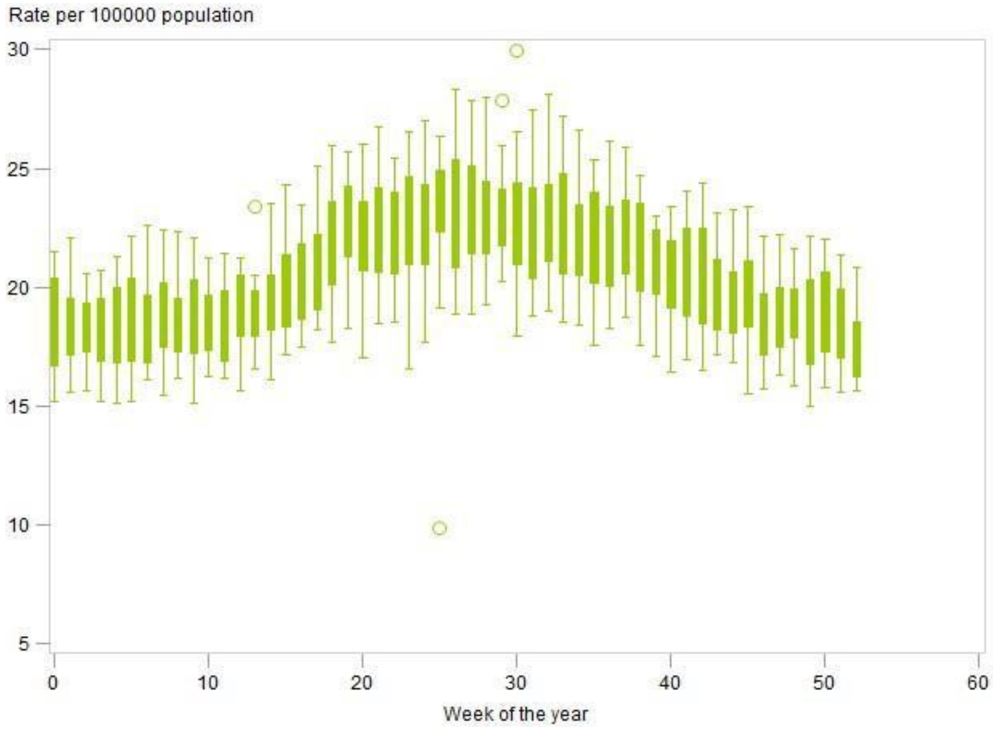

b. 50-64 years

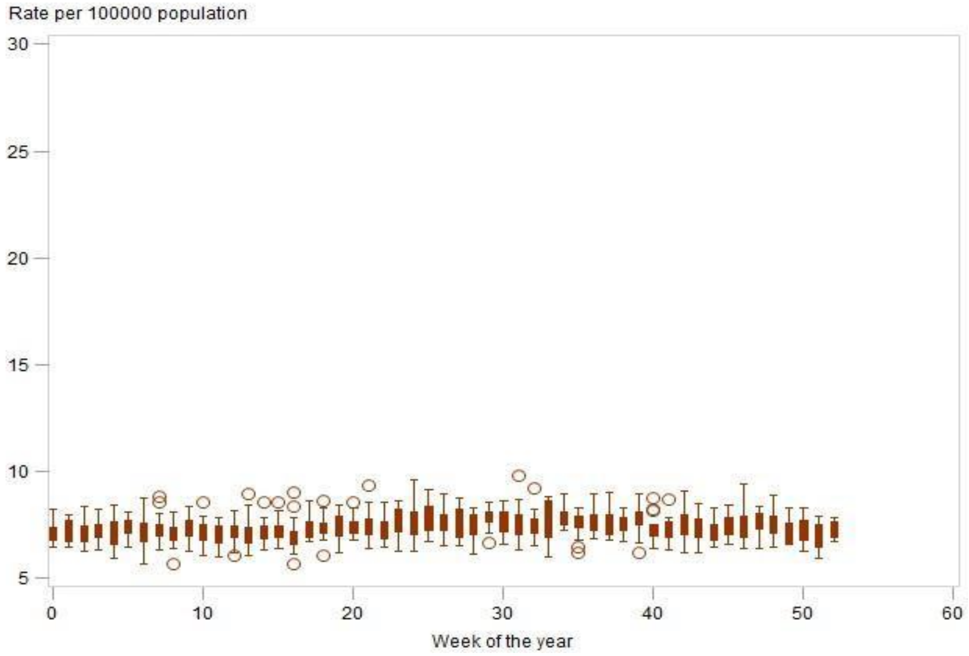

Figure S5. Laboratory-confirmed influenza notifications by type, subtype and year, Australia, 2001-2017.

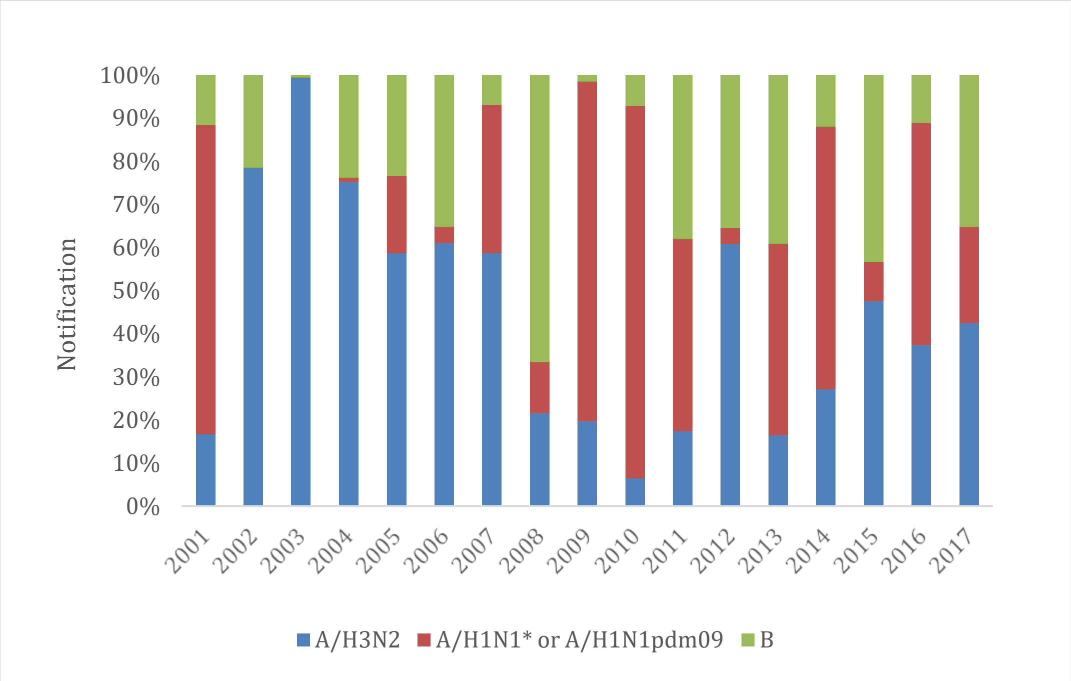

* A/H1N1 strain was replaced by A/H1N1pdm09 from 2009 onwards.

Source: NNDSS (WHOCC)

Hospitalisation

Figure S6: Estimated, observed and baseline hospitalisation rate* of influenza-attributable circulatory and myocardial infraction, by year and age group, Australia, 2001-2017.

a. Circulatory

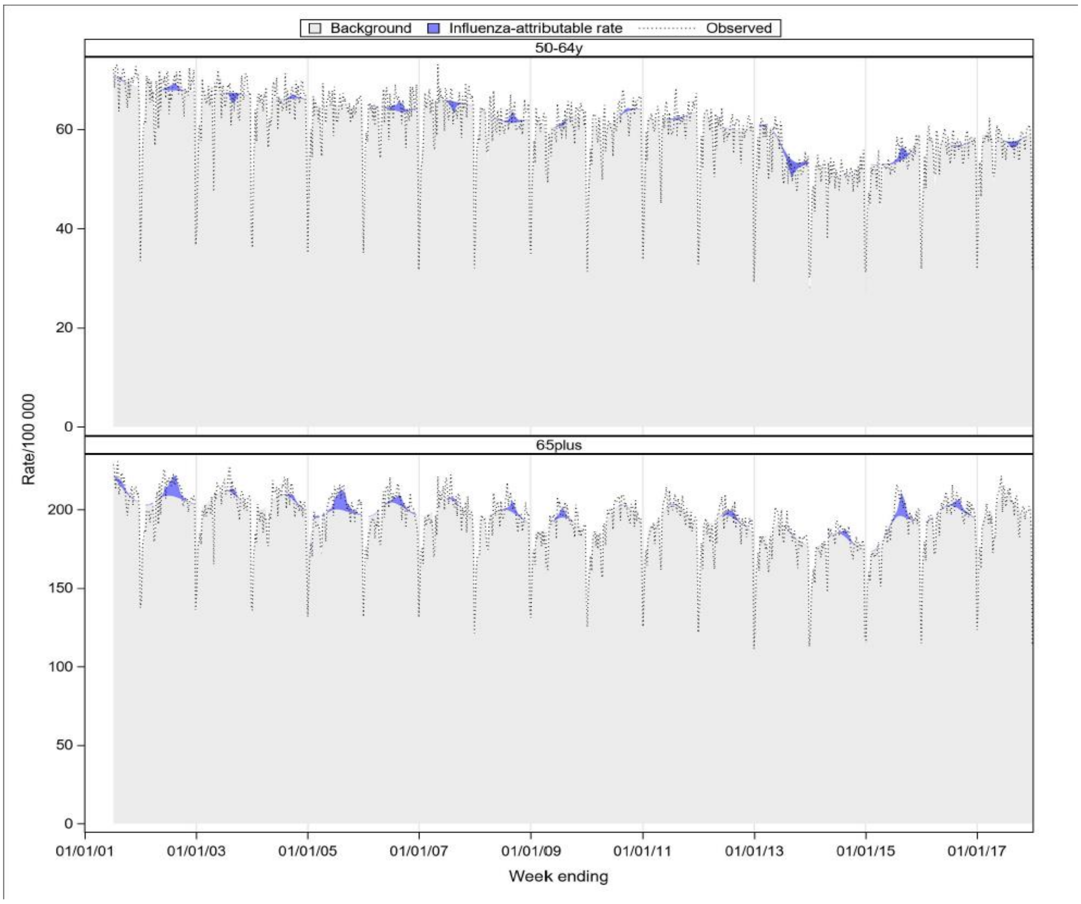

Note: Blue-coloured area indicates rate of influenza-attributable hospitalisation and light grey area denotes baseline hospitalisation rate in the graph.

* Rate per 100,000 population.

b. Myocardial infraction

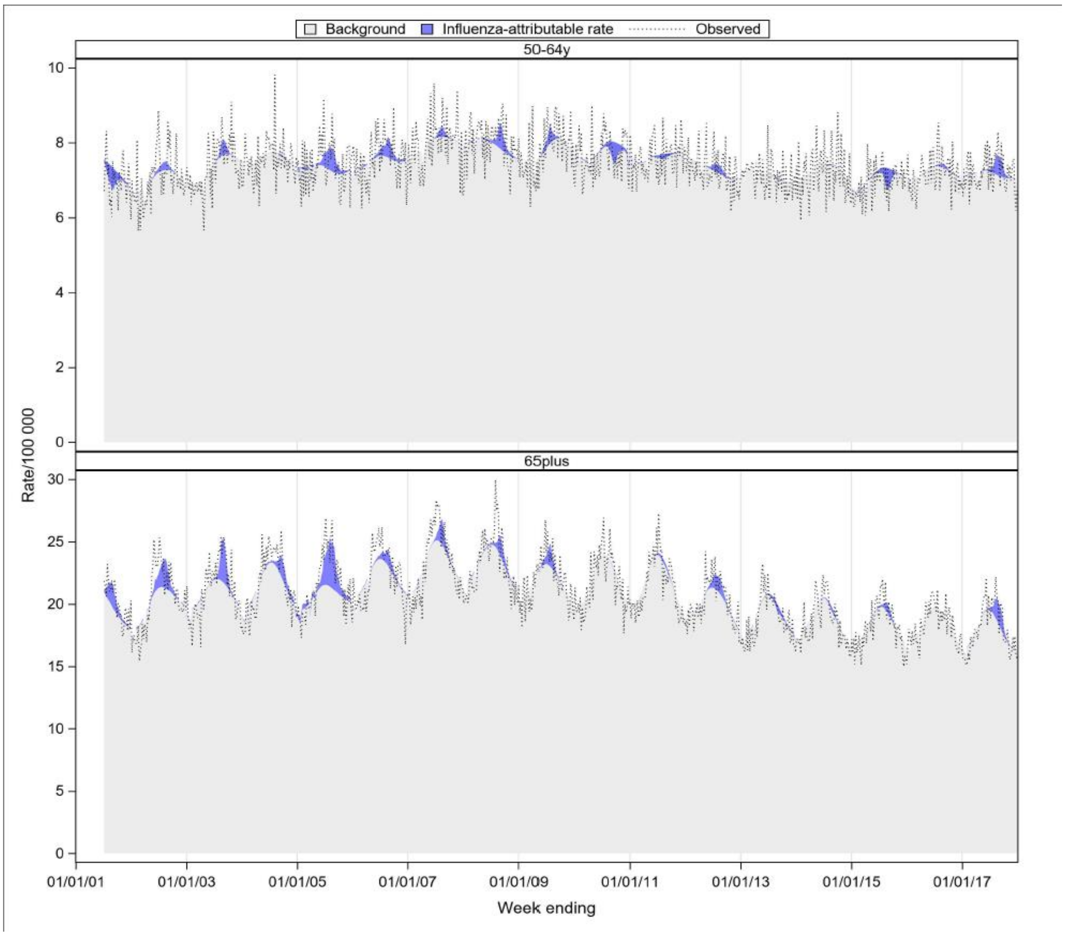

Note: Blue-coloured area indicates rate of influenza-attributable hospitalisation and light grey area denotes baseline hospitalisation rate in the graph.

* Rate per 100,000 population.

Mortality

Figure S7: Estimated, observed and baseline rate* of influenza-attributable influenza/pneumonia and respiratory death, by year and age group, Australia, 2001- 2017.

a. Influenza and pneumonia

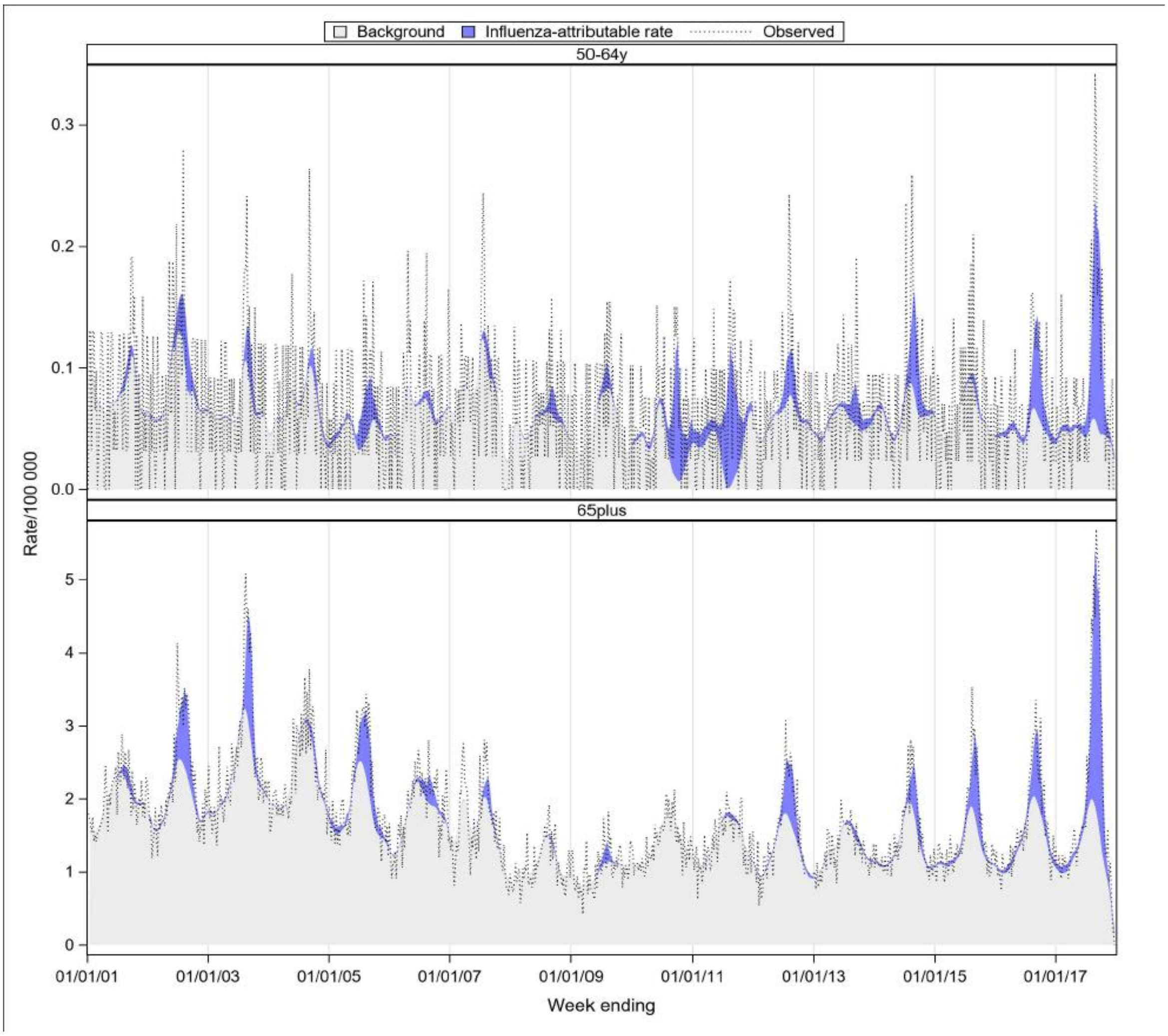

Note: Blue-coloured area indicates rate of influenza-attributable death and light greyarea denotes baseline death rate in the graph.

* Rate per 100,000 population.

b. Respiratory

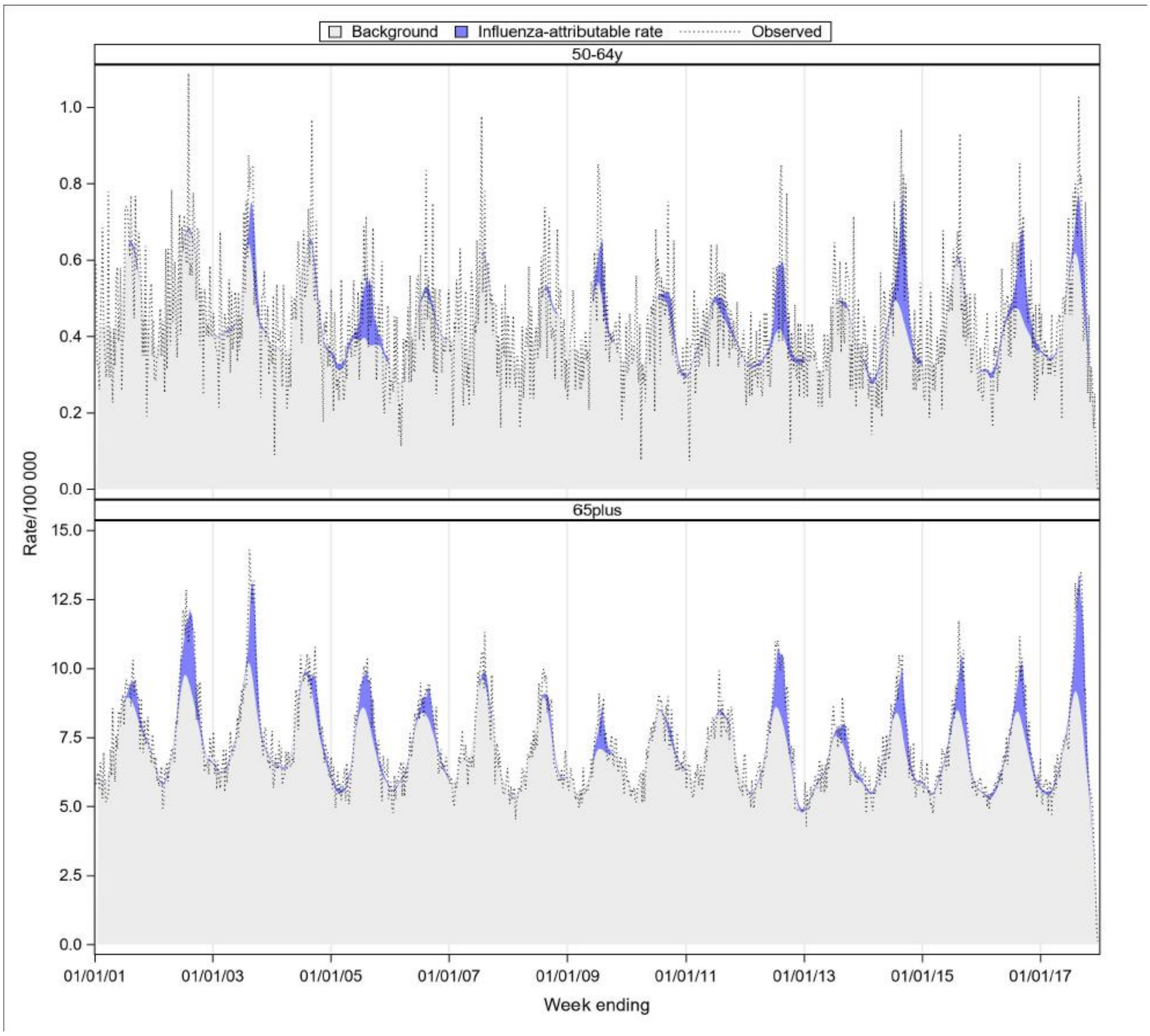

Note: Blue-coloured area indicates rate of influenza-attributable death and light greyarea denotes baseline death rate in the graph.

* Rate per 100,000 population.
